# The internet trade of counterfeit spirits in Russia – an emerging problem undermining alcohol, public health and youth protection policies?

**DOI:** 10.12688/f1000research.11418.2

**Published:** 2017-06-27

**Authors:** Maria Neufeld, Dirk W. Lachenmeier, Stephan G. Walch, Jürgen Rehm

**Affiliations:** 1 Institute for Mental Health Policy Research, Centre for Addiction and Mental Health (CAMH), Toronto, ON, M5S 2S1, Canada; 2 Institute for Clinical Psychology and Psychotherapy, TU Dresden, Dresden, 01187, Germany; 3 Chemisches und Veterinäruntersuchungsamt (CVUA) Karlsruhe, Karlsruhe, 76187, Germany; 4 Dalla Lana School of Public Health, University of Toronto, Toronto, ON, M5T 3M7, Canada; 5 Department of Psychiatry, University of Toronto, Toronto, ON, M5T 1R8, Canada; 6 Institute of Medical Science (IMS), University of Toronto, Toronto, ON, M5S 1A8, Canada

**Keywords:** alcoholic beverages, internet sales, counterfeit, unrecorded alcohol, methanol poisoning

## Abstract

Counterfeit alcohol belongs to the category of unrecorded alcohol not reflected in official statistics. The internet trade of alcoholic beverages has been prohibited by the Russian Federation since 2007, but various sellers still offer counterfeit spirits (i.e., forged brand spirits) over the internet to Russian consumers, mostly in a non-deceptive fashion at prices up to 15 times lower than in regular sale. The public health issues arising from this unregulated trade include potential harm to underage drinkers, hazards due to toxic ingredients such as methanol, but most importantly alcohol harms due to potentially increased drinking volumes due to low prices and high availability on the internet. The internet sale also undermines existing alcohol policies such as restrictions of sale locations, sale times and minimum pricing. The need to enforce measures against counterfeiting of spirits, but specifically their internet trade should be implemented as key elements of alcohol policies to reduce unrecorded alcohol consumption, which is currently about 33 % of total consumption in Russia.

## Unrecorded consumption in Russia

Unrecorded alcohol is alcohol, not reflected in official statistics, but consumed as a beverage ^1^. Since it is untaxed, it is usually much cheaper than regular alcohol ^2^. For Russia, unrecorded consumption is estimated at about 33.4% (5.3 litres of pure alcohol per capita per year) of the consumed alcohol ( 3; for an overview see 4).

Previous studies in Russia have focused on the consumption of alcohol surrogates ^5– 9^ or homemade alcohol ^6, 10, 11^, but comparatively little research has been done on other forms of unrecorded products, such as counterfeit alcohol ^12, 13^. In the course of an ongoing longitudinal study on unrecorded alcohol consumption in Western Siberia ^14^, we have observed various internet sellers of counterfeit spirits, ranging from individual offers on social networks and micro-blogs to specialized online shops offering forged expensive spirits brands.

## The structure and role of internet shops: legal issues

Despite the fact that internet trade of alcoholic beverages was prohibited in 2007 as part of measures to reduce counterfeit sales ^15^, we recorded more than 25 online sellers of counterfeit alcohol, all of which appeared to be maintained and operational (unpublished report). All of the observed platforms were structured similarly, typically featuring a product catalogue, a section about the delivery process and payment, a FAQ section and a contact page; they offered a delivery service against payment, typically for bulk orders only. The prices of the offered products were considerably lower than the regular market prices for retail sale, for example the prices of international vodka brands were 6 times lower than in regular sale, and prices of international spirits such as rum and whiskey were 10–15 times lower. Qualitative interviews revealed that consumers were well aware of the fact, that the beverages offered were not originals ^16^.

## Experiences from the research project

Our first attempt to order online failed. The seller kept the 100% advance payment and never answered our e-mails. The failure to deliver however, may have been the result of a common internet scam rather than a legal issue connected with the official ban on the online trade of alcohol. For the second order a seller with a 50% advance payment scheme was chosen; over 100 bottles of counterfeit spirits were delivered within the following four weeks. One of the ordered international vodkas was not delivered, but instead we received a cheaper Russian brand. Although all of the delivered bottles had Russian excise stamps attached to them, we suspected these were counterfeits because of the low price of the product, and this was later confirmed by chemical analyses. However, the appearance of the bottles was in good accordance with the original products. The accompanying documents of the delivery package indicated payment of freight costs, but not the payment for alcohol itself. Processing payments for the delivery of alcohol is legal according to Russian law and seems to be an important loophole in the existing legislation frequently used by various online sellers, regardless of whether their products are counterfeits or not ^17^.

## Potential health issues of counterfeit alcohol

There are several health issues arising from this unregulated online trade of counterfeit spirits. One problem is the potential harm to health of underage drinkers, a vulnerable group, specifically as the developing brain may be affected ^18^. Undermining of youth protection laws by internet trade because of lack of regulation or its enforcement has been observed in European Union countries as well ^19^.

Since the manufacturing of counterfeits does not follow unified guidelines of product composition and safety, they might contain harmful and toxic ingredients besides ethanol itself. For example methanol poisonings with counterfeit branded spirits have occurred regularly in Russia over the last two years, resulting in several deaths ^14, 20^. These cases corroborate the observed lack of enforcement of food safety standards in internet trade ^21^. However, methanol intoxications are only isolated cases in relation to the general problem of ethanol’s adverse effects. Hence, the main problem with counterfeit spirits lies with their cheap price, high availability and their high demand by certain population groups leading to heavy consumption. Studies suggest that consumption of non-deceptive counterfeits (products of which the consumers are fully aware that they are counterfeits usually because of their cheap price) is associated with consumers of lower socio-economic strata, heavy drinking and consumption of further types of unrecorded alcohol, including alcohol surrogates ^13, 16^. Cheaper alcohol products have been linked to most risky patterns of consumption associated with premature mortality ^22– 24^.

The illegal internet sale of counterfeit alcohol does not only evade tax payments and undermines youth protection policies, it also undermines various other alcohol policy measures introduced in Russia over the last years to reduce the harmful use of alcohol ^25^, such as restrictions of sale locations and sale times and the fixed minimum price on alcoholic beverages ^25, 26^.

## Conclusions

Research in Russia suggests that the Internet has become an important trade channel of counterfeits ^12– 14^ with the observed online sellers apparently operating as an intermediate link in the distribution chain of counterfeit alcohol in Russia, meeting the consumer’s stable demand for cheap alcohol. Therefore, measures against counterfeiting of alcohol should be part of specific policies to reduce unrecorded alcohol consumption and alcohol-related harms in Russia. These measures should include not only legislation, where increasingly higher penalties for the illegal use of trademarks over the past six years were implemented ^27^, but also law enforcement, where the laws seemingly are not really enforced, as the many internet sources for alcohol reveal. In addition, consistent monitoring of the entire production and supply chain of alcoholic beverages as well as effective denaturing of alcohol not intended for human consumption should be considered as the two key elements to achieve this goal ^28^.
