# Supplementary material for: The internet trade of counterfeit spirits in Russia – an emerging problem undermining alcohol, public health and youth protection policies?
Source: F1000Res. 2017 Jun 27;6:520. Originally published 2017 Apr 20. [Version 2] doi: 10.12688/f1000research.11418.2 (PMC5473403; doi:10.12688/f1000research.11418.2)
Supplement: Peer review history

# Peer review: Referee response for version 1

**DOI:** 10.5256/f1000research.12328.r23405

**Published:** 2017-06-12

**Authors:** Yury Evgeny Razvodovsky

**Affiliations:** 1 Grodno State Medical University, Grodno, Belarus

The illegal trade of the counterfeit alcohol is the growing problem in many European countries ^1^. Especially cause worries about recent growth of the infiltration the counterfeit alcoholic beverages into online markets and social media platforms ^1^.

 The paper written by Neufeld and coauthors addresses the relatively new phenomenon, which did not have much space in the scientific literature: the illegal internet trade of the counterfeit alcohol in Russia ^2^. The topic itself is extremely important from a public health perspective, because consumption of alcohol surrogates and counterfeit spirits was identified as one of the major contributor to alcohol-related deaths toll in Russia ^3^^,^^4^. Therefore, this paper is the helpful contribution to fill up this gap. The authors reasonably argue that the internet market of the counterfeit spirits has become an important trade channel of the unrecorded alcohol in Russia, which poses risk to human health due to toxic chemicals, such as methanol; and, also undermines the measures of the alcohol control policy, introduced in this country over the last decade.

 The scale of this problem is well illustrated by the case of the mass poisoning in Krasnoyarsk city (Siberia) in 2015, due to consumption of the falsification whisky bought on the Internet, which killed almost 30 people. According to newspaper reports, over 6.000 bottles of Jack Daniels were seized in this city, which contained a mixture of methanol, ethanol and the water.

 In relation to this, the Russian government should consider a number of potentially effective approaches addressing to the problem of the counterfeit spirits, including raising public awareness of the life treating danger, posed by these products, and also taking the legal actions against the people, offering counterfeit alcoholic beverages for sale on websites and social media platforms.

I have read this submission. I believe that I have an appropriate level of expertise to confirm that it is of an acceptable scientific standard.

## References

[1] EllisC: Organized crime and illicit trade in tobacco, alcohol and pharmaceutical. *RUSI Whitehall Report*.2017;

[2] NeufeldMLachenmeierDWalchSRehmJ: The internet trade of counterfeit spirits in Russia ? an emerging problem undermining alcohol, public health and youth protection policies?. *F1000Research*.2017;6: 10.12688/f1000research.11418.1 10.12688/f1000research.11418.1 28663784PMC5473403

[3] SolodunYVMonakhovaYBKuballaTSamokhvalovAVRehmJLachenmeierDW: Unrecorded alcohol consumption in Russia: toxic denaturants and disinfectants pose additional risks. *Interdiscip Toxicol*.2011;4(4) : 10.2478/v10102-011-0030-x 198-205 10.2478/v10102-011-0030-x 22319254PMC3274728

[4] NemtsovAVRazvodovskyYE: The estimation of the level of alcohol consumption in Russia: a review of the literature. *Sobriology*.2017;

# Response

**Published:** 2017-06-19

**Authors:** Dirk Lachenmeier

**Affiliations:** 42, Chemisches und Veterinäruntersuchungsamt (CVUA) Karlsruhe, Germany

We thank the reviewer for his insight and providing a further example that strengthens our arguments. Regarding approaches, we have added some further suggestions in the revision of our article (see also response to the second reviewer below).

# Peer review: Referee response for version 1

**DOI:** 10.5256/f1000research.12328.r22029

**Published:** 2017-05-03

**Authors:** Andrey V. Korotayev

**Affiliations:** 1 Laboratory for Monitoring Risks of Socio-Political Destabilization, National Research University Higher School of Economics, Moscow, Russian Federation

Notwithstanding the impressive successes achieved by the Russian government and civil society since 2005 in the reduction of the excessive alcohol consumption and the associated harm, the alcohol consumption in Russia still remains very high and constitute the single most important cause of the excessive mortality in this country ^1^. That is why it is so important to identify new measures that could contribute to the further reduction of the alcohol consumption in Russia, and that is why the reviewed article appears so timely and significant. Indeed, the authors demonstrate quite convincingly that the illegal internet sale of counterfeit alcohol undermines various alcohol policy measures introduced in Russia over the last years to reduce the harmful use of alcohol, such as restrictions of sale locations and sale times and the fixed minimum price on alcoholic beverages. 

 The only problem with the article is that its authors do not offer any effective recommendations whose implementation could result in the elimination of the illegal internet sale of alcohol. In fact, they recommend two measures: "consistent monitoring of the entire production and supply chain of alcoholic beverages as well as effective denaturing of alcohol not intended for human consumption". However, I do not think that the authors themselves believe that the implementation of these two measures will result in the elimination of the illegal internet sale of alcohol. Thus, I would suggest that they should think about some additional policy measures. In this respect, it would be very useful if they could study how other countries have managed to solve this problem. In addition, the recommendation to establish "consistent monitoring of the entire production and supply chain of alcoholic beverages" does not appear to be sufficiently specific - the authors should specify what measures should be taken in order to establish such a monitoring.

I have read this submission. I believe that I have an appropriate level of expertise to confirm that it is of an acceptable scientific standard.

## References

[1] KhaltourinaDKorotayevA: Effects of Specific Alcohol Control Policy Measures on Alcohol-Related Mortality in Russia from 1998 to 2013. *Alcohol Alcohol*.2015;50(5) : 10.1093/alcalc/agv042 588-601 10.1093/alcalc/agv042 25964243

# Response

**Published:** 2017-06-19

**Authors:** Dirk Lachenmeier

**Affiliations:** 42, Chemisches und Veterinäruntersuchungsamt (CVUA) Karlsruhe, Germany

Thank you for providing some further background into the issue of alcohol-related harm in Russia.

 As requested, we have added some further aspects on potential measures into the conclusion section. The problem appears to be rather on the enforcement side than on the policy side. For example, higher penalties were recently introduced into the general laws against counterfeiting. Otherwise, we agree with the reviewer that control of internet trade beyond borders is an extremely challenging task and that adequate responses by authorities need to be developed with priority (but such research would be beyond the scope of this 1000-word opinion piece).
